# Aortic Dissection Occurring 18 Months after Successful Endovascular Repair in an Anatomically Difficult Case of Abdominal Aortic Aneurysm

**DOI:** 10.1155/2013/412708

**Published:** 2013-11-04

**Authors:** Satoshi Yamamoto, Katsuyuki Hoshina, Yutaka Takazawa, Hiroyuki Okamoto, Kunihiro Shigematsu, Tetsuro Miyata, Toshiaki Watanabe

**Affiliations:** ^1^Division of Vascular Surgery, Department of Surgery, Graduate School of Medicine, The University of Tokyo, 7-3-1, Hongo, Bunkyo-ku, Tokyo 113-8655, Japan; ^2^Department of Pathology, Graduate School of Medicine, The University of Tokyo, Tokyo, Japan; ^3^Division of Surgical Oncology, Department of Surgery, Graduate School of Medicine, The University of Tokyo, Tokyo, Japan

## Abstract

We report an autopsy case of aneurysm dissection that occurred 18 months after the implantation of a Zenith stent graft. A 94-year-old woman, who had undergone an endovascular repair with postoperative reintervention, died of shock due to retroperitoneal hematoma. An autopsy indicated that the stent graft remained firmly fixed to the native aorta, whereas the dissection occurred near the proximal edge of the stent graft but not at the point of attachment between the suprarenal stent hook and the aorta. The luminal surface of the stent graft was almost completely covered with a transparent film with an endothelial cell lining, which might reflect the tissue regeneration observed on histological examination. This was a rare case of acute aortic dissection that occurred 18 months after EVAR, in which the autopsy indicated interesting microscopic findings and the mechanisms underlying the aortic dissection. We believe that aggressive reintervention at the proximal site in elderly women might cause the dissection of the native aorta.

## 1. Introduction

 Detection of acute aortic dissections during the mid- to long-term followups after endovascular aneurysm repair (EVAR) for abdominal aortic aneurysms (AAAs) is rare. To our knowledge, only 2 cases of aortic dissection after EVAR for AAA and no case involving antegrade dissection and late onset have been reported thus far [1, 2].

 Recently, EVAR has been clinically applied to conditions not included in the instructions for use (IFU) and has been performed even in nonagenarians, with safe and effective outcomes, at least over a short term [3–6]. At our institute, we also perform EVAR for challenging cases as per our previously reported algorithm for the intraoperative management of proximal fixation and have noted good midterm results [7]. Moreover, the short-term outcomes of EVAR have been acceptable; however, clear evidence on the long-term outcomes and durability of the devices is scarce [8, 9].

 Here, we present the case of a high-risk patient with an anatomically complicated AAA, who died of shock due to a retroperitoneal hematoma occurring 18 months after EVAR. We also assessed the histological conditions around the stent graft and discussed the mechanisms leading to the dissection. 

## 2. Case Presentation

### 2.1. Patient History

 The patient was a 94-year-old woman. An AAA with a diameter of 30 mm was incidentally detected by computed tomography (CT); its diameter increased to 55 mm in 6 years. She had previously undergone gastrectomy for gastric cancer and had abdominal incisional hernia; she also had chronic obstructive pulmonary disease and hypertension. Since she was a high-risk and an elderly patient, we decided to perform EVAR. The anatomical conditions of the aneurysm were complex, including a short proximal neck (length: 13 mm), narrow terminal aorta (diameter: 15 mm) showing calcification, and severe calcification of the access route. She underwent EVAR using the Zenith^®^ endovascular graft (Zenith: Cook Medical, IN, USA). Postoperative CT imaging revealed narrowing of the left leg of the stent, poor dilatation of the junction of its right leg, and signs suggestive of type III endoleak (junction leakage) and type I endoleak from the proximal neck. We performed retouchup of the proximal neck with a noncompliant balloon and kissing touchup for the narrow areas and junction; the improvement in the narrowing of the leg and resolution of endoleak were then confirmed by CT. The aneurysm diameter decreased to 41 mm within 1 year. However, after 18 months, she was admitted to our hospital with appetite loss and back pain. Blood tests indicated a hemoglobin level of 6.8 g/dL and a serum creatinine level of 2.46 mg/dL. Plain CT imaging indicated the enlargement of the aneurysm and the presence of a massive retroperitoneal hematoma. Considering these findings, dissection of the aorta at the pararenal site was suspected, and endovascular reintervention was considered impossible under the prevalent conditions. Laparotomy was believed to be a high-risk procedure for the patient, primarily due to her age, and the patient and her family refused further surgical treatment. Instead, she received conservative treatment, but she died 1 day after admission, and an autopsy was performed.

### 2.2. Autopsy

**Figure 1:**
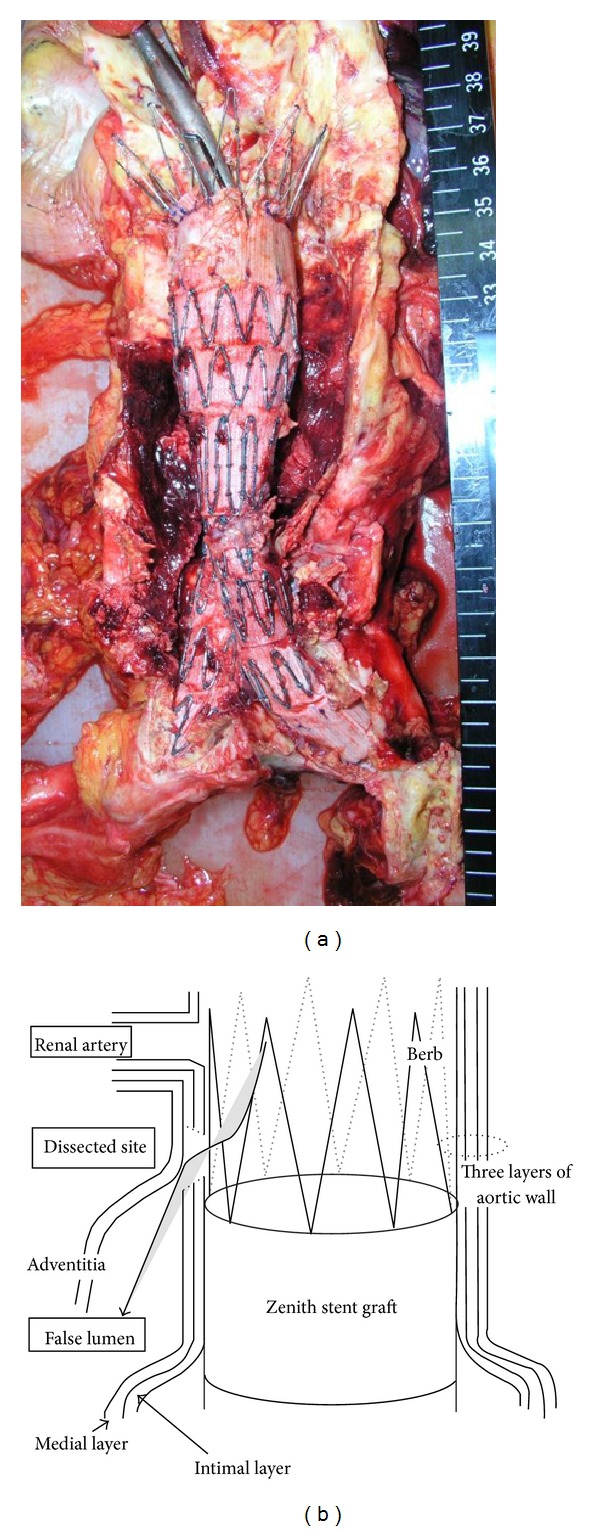
(a) Image of the stent graft after the aneurysmal wall was opened. The stent graft was firmly fixed on the native aortic wall at both the proximal and distal necks. (b) A schema representing the aortic dissection. The dissected site was located just below the right renal artery and the proximal region of the stent graft edge. The device material was fixed firmly to the native aorta.

**Figure 2:**
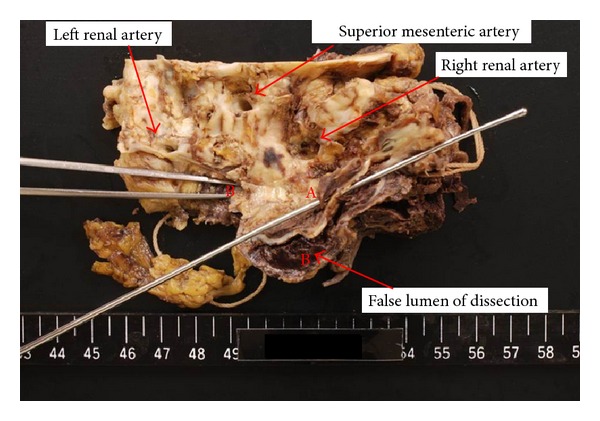
Specimen of the proximal neck after the stent graft was retrieved (view from the luminal side). (a) A tear communicating with the dissected lumen. (b) The false lumen of the dissection.

 Gross examination revealed a massive retroperitoneal hematoma (Figure 1(a)). Additionally, the stent graft remained firmly fixed on the native aortic wall at both the proximal and distal necks, with no space between the stent graft and native aortic wall. A small aortic tear was observed just above the proximal end of stent graft, and the tear communicated with a false lumen between the adventitia and medial layers, resulting in a massive hematoma (Figures 1(b) and 2). The dissection extended from a point just below the origin of the renal arteries to the bifurcation of the common iliac arteries. No fabric tears or stent structure injury were observed, and no dissection was observed at the site of contact between the suprarenal stent hook (barb) and native aorta.

**Figure 3:**
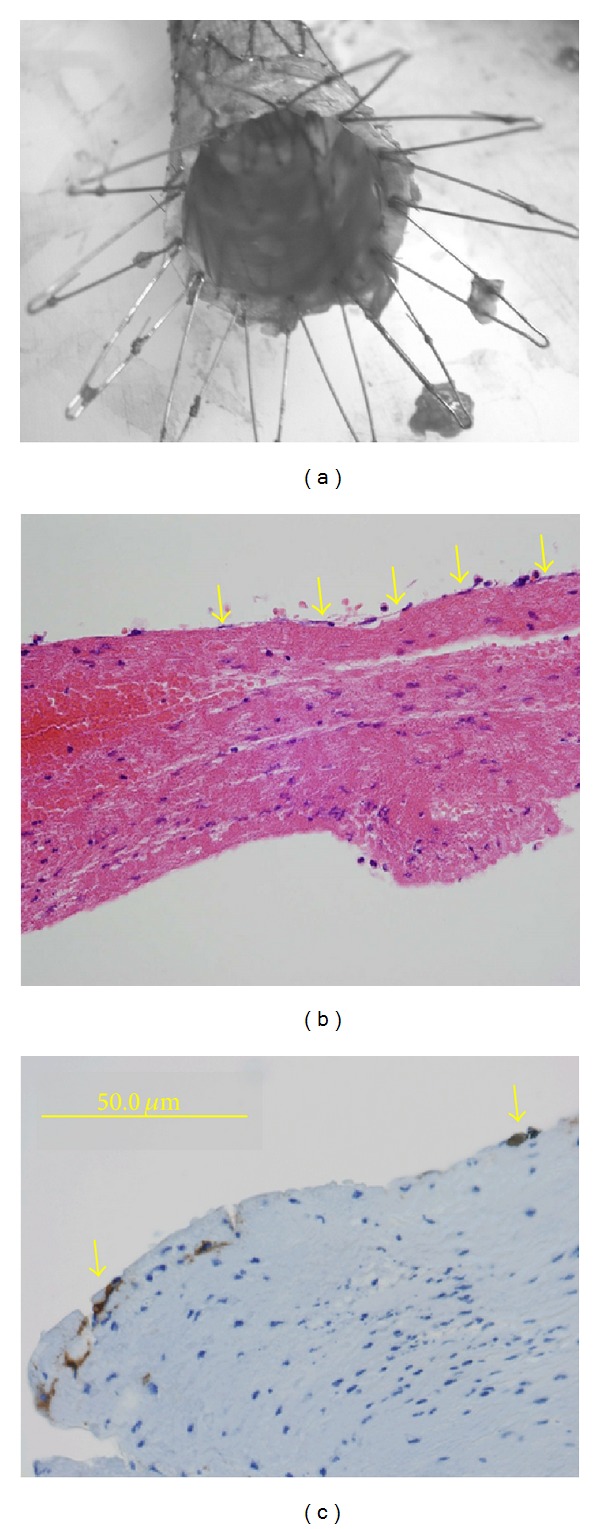
(a) The retrieved stent graft. A thin membranous layer covered the luminal surface of the graft. (b) Microscopic examination indicating the presence of a membranous layer, including cellular components lining on the surface (arrows) (hematoxylin and eosin staining). (c) Endothelial cells were observed on the innermost surface of the layer (arrows) (anti-CD34 antibody staining).

 The inner surface of the stent graft was covered with a transparent glistering film (Figure 3(a)). Microscopic examination indicated the presence of a thin membranous layer composed of accumulated fibrin including deposited red blood cells and infiltrated neutrophils (Figure 3(b)). Moreover, the luminal surface of the stent graft was covered with a lining of endothelial cells (ECs), as confirmed by anti-CD34 staining (Figure 3(c)). 

## 3. Discussion

Since EVAR has shown good outcomes in AAA [8, 10], EVAR is increasingly being used for difficult anatomical cases and conditions violating the IFU of the commercially available devices [3–5]. Despite being beneficial to high-risk patients, the procedure carries certain risks, and the margin of safety should be carefully ascertained because large-scale studies proving the superiority of EVAR over open surgery in long-term outcomes are currently lacking. The long-term complications and questionable durability of the stent graft are problems encountered in this endovascular era.

 Our patient died of acute aortic dissection, which may have developed due to the procedure itself. Although the site of the dissection was not located at the attachment points of both the stent graft and the metallic barb, this adverse event may be a procedure-related, rather than an incidental, complication. 

 During the EVAR procedure in this case, we performed a touchup procedure at the proximal neck with a noncompliant balloon. As per our algorithm, when a type I endoleak is detected or suspected intraoperatively, we perform a touchup procedure, introduce a noncompliant balloon, and use a supportive device, such as a Palmaz^®^ (Cordis, Johnson & Johnson, Japan) stent or aortic cuffs, in sequence [7]. This strategy may be too aggressive for an atherosclerotic aorta; however, we have rarely encountered procedure-related adverse events at the midterm followup of cases treated using this protocol. In this case, the repeated intervention of the proximal fixation might have caused the dissection of the native aorta.

 Previous studies on elderly individuals who underwent EVAR have indicated good short-term outcomes [6, 11, 12]. However, these findings do not justify the indiscriminate use of EVAR in elderly individuals because the procedure-related mortality and complications in the older population are still significantly higher than those in the younger populations [12]. Additionally, female sex has been reported to be a contributing factor for AAA rupture [13, 14]. We believe that these factors could have together contributed to the aortic wall deterioration in the present case. Even if elderly patients appear healthy, it is important to consider that they are at a high risk for developing complications. Further more, we should account for the vulnerability of the native artery in female patients, including the access route. 

 Although retrograde dissection after thoracic stent grafting is reported to occur in approximately 2.4% of the cases [15], only 2 cases of dissection occurring after EVAR for AAA have been reported [1, 2]. However, antegrade dissection with late onset has not been previously reported. The possible causative etiologies for dissection after the implantation have been classified as device-related, procedure-related, and related to natural disease progression [1, 2, 15, 16]. As for device-related complications, the bare springs could have caused the intimal tear [16]; however, this was not applicable to the present case. Balloon dilation (touchup), direct wire injury, and oversizing of the stent graft were implicated as procedure-related factors, and we believe that all these factors potentially contributed to the dissection in this case. 

 Previous reports have indicated poor tissue healing around various stent grafts [17, 18]. In the present case, we found an EC lining on the inner lumen of the Zenith stent graft. To our knowledge, this is the first report indicating the presence of an EC lining inside the stent graft lumen. The commercially available Zenith graft, which is developed using woven Dacron and a unique knitting method, might possess the ability to promote tissue regeneration. If the effect of the Zenith graft on tissue regeneration can be analyzed and its long-term durability proven, it could be preferable for use in younger patients [19].

 In conclusion, we presented an autopsy case of post-EVAR acute aneurysm dissection in a subject implanted with a Zenith stent graft. We believe that aggressive reintervention at the proximal site of the graft in elderly women might result in delayed adverse events.
